# Combined Glucagon-Like Peptide-1 Receptor Agonist (GLP-1RA) and Sodium-Glucose Cotransporter 2 Inhibitor (SGLT2i) Therapy to Restore Fertility in Patients With Obesity, Polycystic Ovary Syndrome, and Incident Type 2 Diabetes

**DOI:** 10.7759/cureus.102358

**Published:** 2026-01-26

**Authors:** Mainak Banerjee, Sujoy Dasgupta

**Affiliations:** 1 Endocrinology, Narayana RN Tagore Hospital, Narayana Health, Kolkata, IND; 2 Reproductive Medicine, Genome - The Fertility Centre, Kolkata, IND

**Keywords:** diabetes type 2, glp-1 receptor agonist, infertility drugs, obesity treatment, polycystic ovary syndrome (pcos), sodium-glucose cotransporter-2 (sglt2) inhibitors

## Abstract

Obesity and type 2 diabetes (T2D) frequently complicate polycystic ovary syndrome (PCOS), exacerbating subfertility. We report two cases of obese women with newly diagnosed T2D and PCOS-related subfertility successfully treated with a combination of a glucagon-like peptide-1 receptor agonist (GLP-1RA) and a sodium-glucose co-transporter 2 inhibitor (SGLT2i). Both patients achieved rapid, substantial weight loss (23% and 10.2%, respectively), leading to menstrual normalization and accidental spontaneous conception within seven months. Medications were discontinued upon confirmation of pregnancy. Both pregnancies were uncomplicated, resulting in healthy live births. Early combination therapy with GLP-1RA and SGLT2i may offer a potent approach to expedite spontaneous conception in this high-risk population. While inadvertent early pregnancy exposure shows no major teratogenic concern based on emerging preliminary studies, immediate discontinuation with transitioning to metformin and/or insulin prior to planning pregnancy remains the clinical standard until robust safety profiles regarding embryotoxicity are established.

## Introduction

The global rise in obesity has led to an increased incidence of type 2 diabetes (T2D) among women of reproductive age [1]. In polycystic ovary syndrome (PCOS), obesity-driven insulin resistance and hyperandrogenism create a significant barrier to ovulation and fertility. High levels of circulating insulin directly stimulate ovarian theca cells to produce androgens, while simultaneously decreasing sex hormone-binding globulin (SHBG), leading to an elevated free androgen index that arrests follicular development and causes chronic anovulation [2]. While metformin and lifestyle modifications remain the standard of care, they often yield modest weight loss that may be insufficient for rapid metabolic and reproductive restoration in morbidly obese patients who face a high psychological and physiological burden from prolonged subfertility [3,4].

Glucagon-like peptide-1 receptor agonists (GLP-1RA) and sodium-glucose co-transporter 2 inhibitors (SGLT2i) possess well-documented metabolic benefits, yet their combined first-line use for fertility restoration remains under-reported. This case series highlights the additive benefits of this combination in achieving rapid biochemical androgen reduction and spontaneous conception, while addressing the safety concerns regarding inadvertent early pregnancy exposure.

## Case presentation

Case 1

**Figure 1:**
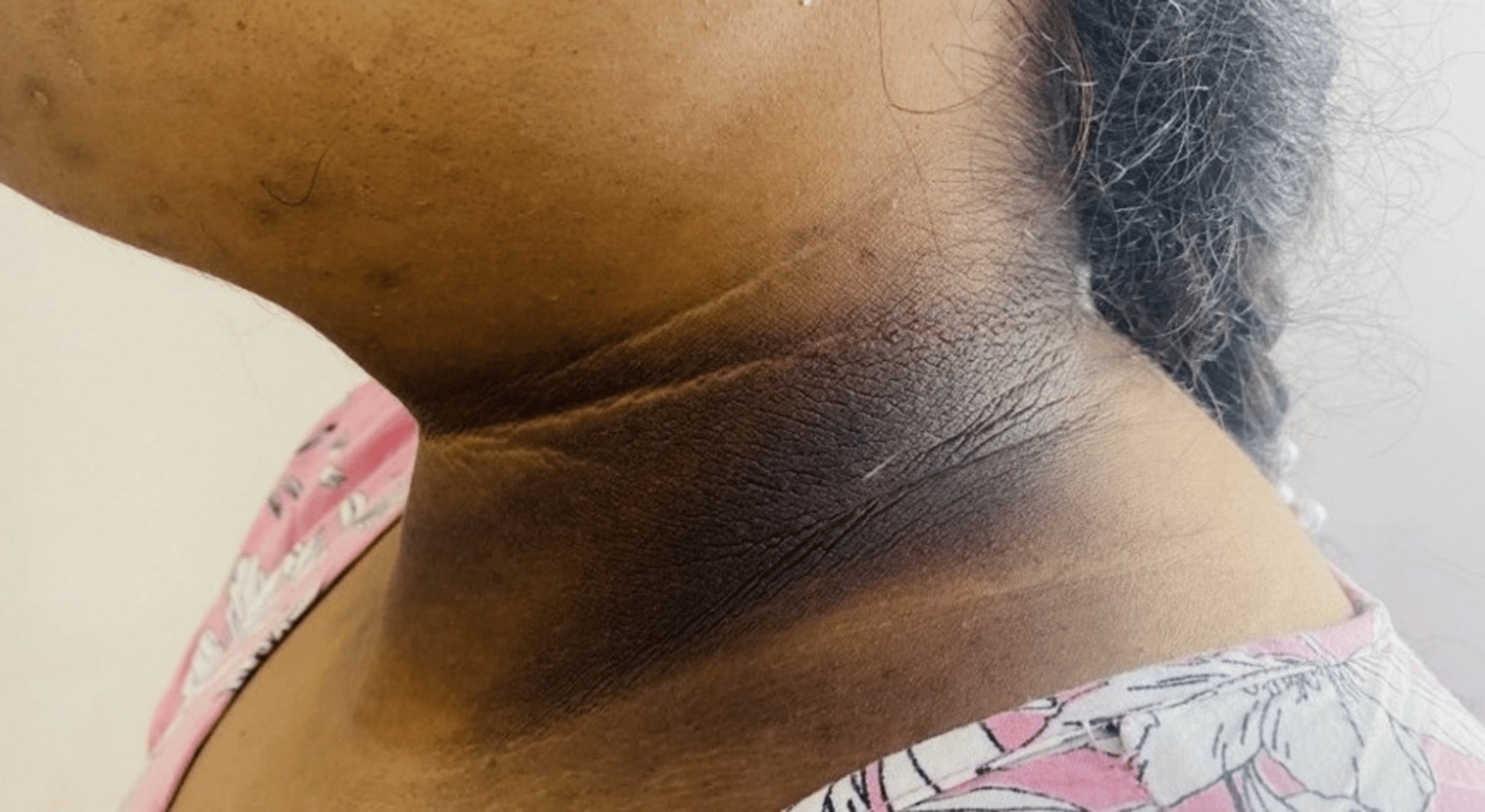
Grade 4 acanthosis in the first patient

A 26-year-old female presented with an 18-month history of primary subfertility and secondary amenorrhea for the last six months. Her medical history included obstructive sleep apnea (OSA) and hypothyroidism. She had previously failed multiple lifestyle-based weight loss attempts and had no history of prior treatment with ovulation-induction agents (e.g., letrozole or clomiphene). Physical examination revealed grade 4 acanthosis nigricans (Figure 1) and a body mass index (BMI) of 47.9 kg/m^2^ (body weight 127.5 kg). Laboratory investigations confirmed newly diagnosed T2D (HbA1c 9.1%, fasting blood glucose (FBG) 155 mg/dL), and metabolic dysfunction-associated steatotic liver disease (MASLD). Her total testosterone, measured using electrochemiluminescence immunoassay, was 51 ng/dL (reference range: 6-70 ng/dL). Ultrasound confirmed polycystic ovarian morphology (volumes: right 10 cc, left 13 cc), fulfilling Rotterdam criteria for PCOS.

**Figure 2:**
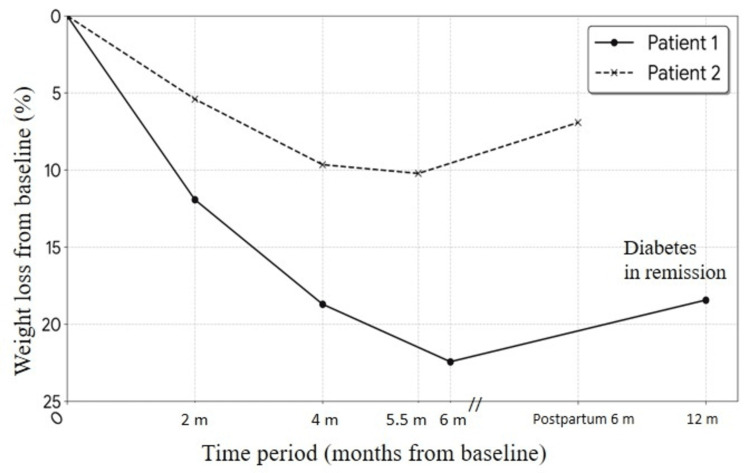
Weight trajectory from baseline

Therapy was initiated with subcutaneous dulaglutide (titrated to 1.5 mg weekly), dapagliflozin 10 mg daily, and metformin 500 mg. Metformin was later discontinued due to dizziness (FBG 76 mg/dL). After six months, the patient achieved a 23% weight reduction (Figure 2) and excellent glucose control (HbA1c 5.6%). Notably, her total testosterone decreased to 23 ng/dL. OSA symptoms improved, and liver enzymes normalized. In the seventh month, she had an accidental spontaneous conception. All study medications were discontinued immediately. The pregnancy progressed without complication, resulting in a healthy 2.9 kg infant. One year postpartum, she remains in T2D remission without pharmacotherapy despite a weight increase to 106 kg (Figure 2).

Case 2

A 28-year-old female presented with a 12-month history of infertility and oligomenorrhea (cycles 40-45 days). Examination revealed a BMI of 36.25 kg/m^2^ (body weight 88.1 kg) and grade 2 acanthosis nigricans. Laboratory tests showed an HbA1c of 7.2% and total testosterone of 48 ng/dL. Ultrasound confirmed polycystic ovarian morphology and grade 2 fatty liver.

She was treated with oral semaglutide (titrated to 7 mg daily) and dapagliflozin 10 mg daily. Over four months, her menstrual cycles normalized following an 8.5 kg weight loss. She conceived spontaneously at 5.5 months after achieving a total weight loss of 10.2% from baseline (Figure 2). Medications were discontinued upon a positive pregnancy test. The pregnancy required insulin starting at 24 weeks, but otherwise remained uncomplicated, resulting in the birth of a healthy 2.6 kg infant.

## Discussion

This series demonstrates that the significant weight loss achieved through an early combinational pharmacotherapy, involving standard glycaemic doses of GLP-1RAs and SGLT2i, can swiftly restore the hypothalamic-pituitary-ovarian axis in morbidly obese women with T2D. The rapid "hyperresponse" may stem from a high degree of underlying metabolic derangements present at baseline [5]. Consequently, the magnitude of weight loss significantly exceeded the 5-10% threshold typically cited for restoring ovulation in PCOS [2,4], likely providing a more rapid “metabolic reset” by simultaneously lowering insulin-driven androgen production and caloric load. The synergy between these agents is likely multi-factorial: GLP-1RAs suppress appetite and enhance satiety, while SGLT2i induces a caloric deficit via glycosuria [6]. While weight loss is a primary driver, the direct complementary insulin-sensitizing effects of this combination also likely address the core pathophysiology of PCOS by reducing insulin-driven ovarian androgen production and systemic inflammation [7,8].

A significant finding in these cases was the lack of macrosomia or adverse maternal outcomes, despite the known risk of rebound weight gain following GLP-1RA cessation. Unlike a previously reported case where substantial gestational weight gain following GLP-1RA discontinuation resulted in fetal macrosomia [9], our patients maintained healthy fetal growth parameters. The findings from the first patient further suggest that the significant metabolic “reset” achieved prior to conception may be sustainable with close monitoring, though it underscores the necessity of clinical vigilance to prevent compensatory overeating or metabolic relapse during the gestational period. As a report of two patients, these findings cannot establish generalizability. It remains difficult to isolate whether fertility restoration was driven primarily by the absolute weight loss, the specific pharmacological action on insulin pathways, or a combination of both.

Another critical concern in this population is inadvertent drug exposure during the first trimester. Animal studies suggest that SGLT2i-related risks (such as renal pelvis dilatation) are primarily late-trimester concerns [10]. Similarly, GLP-1RA-associated fetal risks in animal models often stem from maternal malnutrition due to continuous GLP-1RA use rather than direct teratogenicity [10,11]. Recent human observational data have not shown a significantly increased risk of major congenital malformations following early-pregnancy exposure to these agents compared to other second-line antidiabetics [12]. Nevertheless, transition to established therapies like insulin remains mandatory immediately before pregnancy planning.

## Conclusions

Combined GLP-1RA and SGLT2i therapy may serve as an effective “metabolic bridge” to spontaneous conception for obese women with T2D and PCOS. This approach may potentially reduce the need for invasive and costly fertility treatments, but further observational studies or randomized controlled trials are required to validate efficacy and safety on a larger scale. Moreover, until definitive safety profiles regarding embryotoxicity are established, strict protocols for discontinuation immediately prior to planning conception are essential. Furthermore, clinicians should proactively manage the risk of rebound weight gain throughout the gestational period to maintain maternal and fetal health.

## References

[1] American Diabetes Association Professional Practice Committee (2025). 8. Obesity and weight management for the prevention and treatment of type 2 diabetes: standards of care in diabetes-2025. Diabetes Care.

[2] Teede HJ, Tay CT, Laven JJ (2023). Recommendations from the 2023 international evidence-based guideline for the assessment and management of polycystic ovary syndrome. J Clin Endocrinol Metab.

[3] Yerevanian A, Soukas AA (2019). Metformin: mechanisms in human obesity and weight loss. Curr Obes Rep.

[4] Goldberg A, Graca S, Liu J (2024). Anti-obesity pharmacological agents for polycystic ovary syndrome: a systematic review and meta-analysis to inform the 2023 international evidence-based guideline. Obes Rev.

[5] Jayasinghe KNU, Greener VJ, Feher MD (2016). Combining SGLT2 inhibitor and GLP-1 agonist: exaggerated weight loss in a morbidly obese patient with type 2 diabetes. Br J Diabetes.

[6] Elkind-Hirsch KE, Chappell N, Seidemann E, Storment J, Bellanger D (2021). Exenatide, dapagliflozin, or phentermine/topiramate differentially affect metabolic profiles in polycystic ovary syndrome. J Clin Endocrinol Metab.

[7] Reppo I, Jakobson M, Volke V (2023). Effects of semaglutide and empagliflozin on inflammatory markers in patients with type 2 diabetes. Int J Mol Sci.

[8] Ameho S, Klutstein M (2025). The effect of chronic inflammation on female fertility. Reproduction.

[9] Skov K, Mandic IN, Nyborg KM (2023). Semaglutide and pregnancy. Int J Gynaecol Obstet.

[10] Muller DR, Stenvers DJ, Malekzadeh A, Holleman F, Painter RC, Siegelaar SE (2023). Effects of GLP-1 agonists and SGLT2 inhibitors during pregnancy and lactation on offspring outcomes: a systematic review of the evidence. Front Endocrinol (Lausanne).

[11] Goldberg AS, Boots CE (2024). Treating obesity and fertility in the era of glucagon-like peptide 1 receptor agonists. Fertil Steril.

[12] Cesta CE, Rotem R, Bateman BT (2024). Safety of GLP-1 receptor agonists and other second-line antidiabetics in early pregnancy. JAMA Intern Med.

